# The Selection of Resident Medical Officers

**Published:** 1907-05-11

**Authors:** 


### Resident Medical Officers' Department.

### THE SELECTION OF RESIDENT MEDICAL OFFICERS.

It is not out of place to consider in this section some of the points which influence hospital committees and visiting staffs in the appointment of men to the posts of house officers. We must admit at the outset that some of the following remarks are based upon hearsay, and that others are merely conjectural. No one outside the selection committee itself ever really knows for certain upon what grounds the house appointments in a particular hospital are made. But it is quite possible to form some estimate of the broad general rules which may be supposed to govern such selections in the majority of institutions, and it is desirable that any details that are known should be discussed and criticised.

Merit, and merit alone, is of course the one criterion, and the only recommendation with all such committees. That, at least, is the *ex parte* statement which they themselves supply; and we must accept it in the hope that it is true. Any suggestion of favouritism is hotly denied. The only ideal which they profess to strive after is the appointment of the best man for the post. But what exactly constitutes merit in a prospective house officer they are not quite so willing to make public; and those who divulge their standards of judgment in this matter show the greatest diversity of opinion and of method.

With some selection committees a brilliant academic record weighs heavily; with others scholarships, prizes, and medals count for nothing. With some, personal appearance, manners, and manner carry the day; others look askance at these ornaments, and consider roughness and *gaucherie* the hall-mark of a keen, hard-working, practical man. So, too, with testimonials and personal recommendations; some committees are greatly influenced by them, while others laugh them to scorn. So many men, so many opinions. On what points, then, do they agree, if indeed they agree on any points at all?

In the first place it may reasonably be supposed that every impartial hospital committee wishes to have resident medical officers who are not only capable, enthusiastic and conscientious, but who are also level-headed and likely to maintain pleasant relations with their superior officers, and with the nursing and administrative staffs—practical, strong-minded fellows, kind to their patients, popular with their colleagues, capable of doing good work themselves and of getting the best work out of other people. These are the men who advance the prestige of any institution to which they attach themselves, and such as these that every selection committee is eager to obtain.

In the second place, the authorities of a hospital naturally desire for the posts of house officers young men of good physique, inured to the close and vitiated atmosphere of hospitals, and possessed of sufficient adaptability and endurance to come through the ordeal of continuous hard work and responsibility, by night and by day, without breaking down or even complaining.

Few people are likely to cavil at this picture of the ideal house officer. But it is not so easy for us to lay down rules by which he may be discerned among the applicants for a resident house appointment, nor is it easy to criticise with justice the various methods which are at present adopted by committees in their endeavour to find him.

In large hospitals, with medical schools attached, the selection is mainly in the hands of the visiting staff, the men under whom the resident officers are destined to work. The applications are referred to them, and after consultation among themselves they make their nominations, and these are sent to the governors to be confirmed. Having known the applicants as students, and having had ample opportunities of gauging their capacities, and of forming a just estimate of their characters, they are in the best position for making a satisfactory choice.

At many of these hospitals, when (as usually happens) a number of vacancies occur together, each candidate for office has to specify under which physician or surgeon he wishes to act, and each physician or surgeon makes his own choice, without interference from his colleagues, and without in his turn interfering with his colleagues' selections. Thus at these institutions a student who wishes to become, say, a house surgeon has to establish a claim upon one particular surgeon—the one under whom he wishes to serve, and by whom (if successful) he will be nominated. This method has it merits and its demerits. The points in its favour are that each member of the visiting staff obtains as his lieutenant a man personally known to him, whose work and character he has been able to investigate, and who is familiar with his own methods and peculiarities—in other words, it is his own fault if his hospital work is not done satisfactorily and with a maximum of pleasure. On the other hand, this method is open to abuse, for under it a visiting physician, for example, is theoretically at liberty to appoint as his house physician an incompetent man whom he likes, in place of a far better worker whom he happens to be prejudiced against; and the latter, by putting all his eggs into one basket, often loses his chance of a house appointment at his own hospital altogether. A further disadvantage of this system is that it tends to encourage time-serving, toadying, and other specious practices among students, and leads some men to neglect their all-round education in their desire to show up well in a particular teacher's eyes.

But, as a matter of fact, this method, in spite of the theoretical objections that have been raised against it, works very well in practice. It breeds far less favouritism than would be supposed; and, although a deserving man is now and then supplanted by an obvious inferior, the visiting staffs of these hospitals are generally justified in their selections. The subsequent history of a case of alleged nepotism often shows that the best man for the post has after all been chosen.

In one at least of the great Metropolitan hospitals this system is not ostensibly in force. There, a student at the close of each pupil's appointment or course of instruction, from the day he enters to the day he qualifies, receives marks from his teachers; and these marks are systematically recorded. The marks are given on three separate counts—for industry, for ability, and for conduct. If after qualification he applies for a testimonial or a house appointment, these marks are added up and their details are inspected, and his application is supposed to be considered solely from this point of view. It is a method which appears to have much to recommend it. If it is properly carried out, favouritism and jobbery should be reduced to a minimum.

In another hospital, we understand that the junior staff appointments, whenever there are more applicants than there are vacancies, are allotted after a competitive examination. This does not strike us as a sound method, whether the examination is merely nominal, and is conducted as an excuse for the senior staff to reject men whom they do not wish to appoint, or whether it is a genuine means of selection. The ordinary hospital test-papers and the professional examinations, together with the teacher's knowledge of their pupils, should be enough, without piling up further formal tests of book-knowledge and examination skill.

In hospitals not associated with medical schools the vacancies are usually notified by public advertisement in the professional journals. Applicants are invited to send testimonials, and, when required, to appear before the board. As a rule the selection is here again left largely to the honorary medical staff, whose advice in this matter carries considerable weight with the lay members of the committee. Sometimes, however, the lay members assert themselves, and it is then that the glowing testimonial and the gilded hospital record have their opportunity. The non-medical mind hardly as yet realises that a large proportion of medical testimonials are for all practical purposes the merest rubbish. If all these panegyrics are to be taken as sworn statements of attested facts, it follows that 99 per cent. of newly qualified practitioners are the most accomplished and proficient beings that the world has ever seen. As a matter of fact this is not the case, and it is better for lay committees to leave the interpretation of testimonials to their medical colleagues.

No one of these systems is perfect; but on the whole it may be said that, whatever the means of selection, the man who is best fitted for a house appointment, whether in his Alma Mater or in a small provincial hospital, is generally the man who makes the most favourable impression on the majority of the members of the selection committee, lay as well as medical. Under no system will it ever be possible to exclude the personal element from having some influence in the selection of candidates of approximately equal merit. Selection committees are but aggregates of human beings; and a pleasant expression and a decided manner are always likely to turn the scale in a man's favour, when, as often happens, there is little else to distinguish him from his competitors.

